# Task Shifting, eHealth and Shared Decision‐Making—Preference Heterogeneity in the Adult Population for Developments in Outpatient Primary Healthcare

**DOI:** 10.1111/hex.70060

**Published:** 2025-02-05

**Authors:** Zora Föhn

**Affiliations:** ^1^ Faculty of Humanities and Social Sciences University of Lucerne Luzern Switzerland

**Keywords:** discrete choice experiment, health service research, outpatient primary healthcare, preference heterogeneity, public preferences, trends in healthcare

## Abstract

**Introduction:**

Considerable changes in primary outpatient healthcare can be expected in the coming years due to various trends observed in Western countries, such as an ageing population, an increasing number of patients with chronic illnesses, GP shortages, a trend towards patient‐centred care and the digitalization of healthcare. Knowing the population's preferences and subgroup preferences in these developments helps policymakers make healthcare delivery more responsive to patients' needs.

**Methods:**

Primary data were collected using a representative sample of the adult Swiss population in the fall of 2020. Discrete Choice Experiments (DCEs) were conducted, where one of the DCEs included an acute health problem and another a routine examination. In the survey, respondents were shown five different choice tasks, each containing two hypothetical alternatives for primary outpatient healthcare models. They were asked to choose their preferred alternative for each choice task. The DCEs enabled the measurement of the population's preferences for nine attributes of primary outpatient healthcare that are currently under discussion (e.g., task shifting, new technology in healthcare and shared decision‐making). The data were analysed using panel latent class logit models (LCA) to determine preference heterogeneity and relative importance scores for the attributes.

**Results:**

Data from 4745 respondents were included in the analysis. The LCA indicated two classes for acute health problem and three classes for routine examination, with varying latent preference structures. Both DCEs showed similar preference patterns: for one class with younger and more educated respondents, health insurance premium development was most important, followed by participation in decision‐making, whereas having a doctor as the health professional was less important. Another class with older and less educated people attached more importance to personal continuity of care and the health professionals being a medical doctor.

**Conclusion:**

Younger and more highly educated people are more open to current developments in outpatient primary healthcare, such as task shifting, than older and less educated people, especially if this reduces their health insurance premiums in return.

**Patient or Public Contribution:**

The public was involved in the design of the DCE and as the respondents of the survey used in this article.

## 1 Introduction

Considerable changes in primary outpatient healthcare can be expected in the coming years due to various trends observed in Western countries: the proportion of older people is increasing [1], leading to a rising proportion of patients with chronic diseases. This trend is associated with increased use of outpatient primary healthcare and added pressure on the healthcare system [2]. Considering this—and to improve health outcomes, avoid hospital admissions and improve coordinated care—new roles such as coordinating health professionals are being introduced [3]. At the same time, many Western countries are dealing with shortages of general practitioners (GPs), leading to the development of non‐medical health professionals taking over tasks traditionally performed by GPs [4, 5]. Technology, such as electronic patient dossiers, telemedicine or online triage [6], is increasingly pivotal in healthcare, and shared decision‐making is gaining importance in the context of patient‐centred care [7]. These changes (e.g., coordination of healthcare professionals, task shifting and online triage) are also being discussed regarding the rapidly rising healthcare costs in Switzerland, where this study was carried out, with the main aim of slowing down the rise in costs for compulsory health insurance [1, 3, 4, 5].

Knowing the population's preferences in these developments helps policymakers make healthcare delivery more responsive to patients' needs [8]. A prevalent quantitative technique for determining preferences in healthcare is the Discrete Choice Experiment (DCE) [9]. DCEs were previously used to measure the population's preferences or specific patient groups' preferences for different aspects of primary healthcare services. Those DCEs focus mostly on the status quo rather than trends in primary care services [10] or single developing aspects such as patient involvement [11], out‐of‐hours health services [12] and wider nursing roles [13, 14]. This precludes a comparison of relevance between different aspects. Additionally, there is evidence that preferences for adaptions in outpatient primary healthcare are not homogeneous but differ between subgroups of the population [15, 16, 17]. A key determinant of the effectiveness of health policy may be influenced by considering subgroup preferences [18].

Therefore, this study has the following objectives: first, to estimate the preferences of the general adult population for current developments in the primary outpatient healthcare system; second, to provide quantitative information about the relative importance score of different aspects of these developments; and, third, to study possible preference heterogeneity and what characteristics of the population are significantly associated with belonging to a latent preference subgroup.

## 2 Methods

### 2.1 The DCE

The DCE method presumes that any good or service can be described by its characteristics and that the assessment of the good or service by an individual depends on the specification of these characteristics [19]. In a survey, respondents are asked to choose between different hypothetical alternatives described by characteristics (called attributes) that can take on various forms (called attribute levels) [20]. For this study, the alternatives consist of hypothetical primary outpatient healthcare models. An example of an attribute in the study is the opening hours, which can take on different attribute levels such as office hours, extended office hours or around the clock. On the basis of the random utility framework, it is assumed that individuals are rational decision‐makers, presumed to choose the alternative that generates the highest utility for them by trading all attributes of the choice task in their decision‐making [21]. The choices are then analysed to estimate preference weights from which importance scores for the attributes and attribute levels can be estimated and ranked [22, 23].

### 2.2 Design Process of the DCEs

In the first step, the development of a DCE includes selecting the attribute and attribute levels [24]. The starting point for this study's attributes and attribute level selection was a literature analysis [25]. The list of attributes generated through the literature analysis was discussed and supplemented by six one‐on‐one expert interviews and one focus group discussion with eight health science students.

In the second step, a manageable list of possible attributes had to be selected to decrease the respondents' cognitive burden and ensure that they can consider all attributes in their choices [26, 27, 28]. For this purpose, the collected attributes were prioritized according to their relevance by 29 representatives of the general population and nine experts from the field of outpatient primary healthcare. Nine attributes were selected on the basis of these prioritizations (Table 1). Due to their typology, the nine attributes were divided into two DCEs: one of the DCEs included an acute health problem and the other included a routine examination, covering two of the most common reasons for the general population to use the primary outpatient healthcare system. Each of the DCEs consisted of seven attributes. Five attributes are consistent between the two DCEs and two differ, as they are more relevant for one of the situations (Table 1). To reflect the diversity of urgency and uncertainty in consultation with the primary care provider, the respondents were asked to imagine either having a middle ear infection or a sprained ankle when answering the DCE for the acute health problem and, respectively, having a preventive screening or routine examination for chronic diabetes when answering the DCE with a routine examination. The pretest for the survey was conducted in two steps: first, the DCE was pretested through a think‐aloud protocol with six people from the general adult population [24]. Subsequently, the survey was tested in an online pilot with an access panel of 400 Swiss adult residents.

**Table 1:** Attributes and attribute levels used for the DCEs.

DCE	Attributes	Attribute levels
Acute health problem	Health professional/application for first contact	General practitioner
		Pharmacist
		Registered nurse
		Healthcare assistant
		Online application
Acute health problem	Type of first contact	In persona
		Via videophone
		Via phone
Acute health problem/routine examination	Treating health professional	General practitioner
		Specialized medical practitioner
		Registered nurse
		General practitioner via videophone while a registered nurse carries out instructions onsite with the patient
Acute health problem/routine examination	Opening hours	Office hours (9 am−6 pm)
		Extended office hours (7 am−10 pm)
		Around the clock
Acute health problem/routine examination	Continuity	Person providing treatment knows the patient's health history and has access to medical records
		Person providing treatment does not know the patient's health history but has access to medical records
		Person providing treatment does not know the patient's health history and has no access to medical records
Acute health problem/routine examination	Decision‐making regarding treatment	Patient lets health professional decide
		Decision is up to the patient
		Patient and health professional decide together
Routine examination	Coordinating health professional	General practitioner
		Pharmacist
		Registered nurse
		Healthcare assistant
Routine examination	Assignment of the coordinating health professional	Patient chooses himself/herself
		Assigned by the health insurance provider
Acute health problem/routine examination	Development of the monthly health insurance premium compared with today^a^	Increases by 100 Swiss francs
		Increases by 50 Swiss francs
		Stays the same
		Decreases by 50 Swiss francs
		Decreases by 100 Swiss francs

^a^ Compared with the monthly health insurance premium that the individual pays today. Premiums for compulsory basic health insurance in Switzerland are calculated on a per capita basis and vary according to the insurance company, the deductible chosen, the insurance model and the region of residence.

### 2.3 Design of the Study and Survey Format

The second step in designing a DCE is the selection of the experimental design and choice set construction [24]. Combining all the attribute levels would result in an unfeasible number of hypothetical alternatives, given the chosen attributes and attribute levels. Thus, a fixed effects efficient design was used by maximizing D‐efficiency. A D‐efficient design draws a sample of all possible level combinations, which still enables reliable variable estimates [29]. Eventually, on the basis of the number of chosen attribute levels for each DCE, 45 choice sets for the DCE with an acute health problem and 40 choice sets for the DCE with a routine examination were created and divided into nine blocks for the DCE with an acute health problem and eight blocks for the DCE with a routine examination. The experimental design of the choice set was performed using Ngene Software, 1.2.1 [30]. In the survey, the respondents were shown five different choice tasks, each containing two alternatives. The number of tasks and alternatives was chosen with regard to statistical efficiency and minimizing the cognitive burden for the respondents. An example of a choice set included in the study is shown in Supporting Information S1: Appendix A. Three different colours were used in the choice sets as cognitive help for the respondents to distinguish the attributes for the first contact/coordination, main treatment and cost. The survey contained further questions about demographics and health literacy, questions concerning the respondents' satisfaction with current healthcare and additional questions regarding preferences for various organizational aspects of (future) healthcare delivery.

### 2.4 Data Collection

For the data collection, 12,234 adult habitants of Switzerland were randomly selected from the sampling frame of the Swiss Federal Statistical Office. The sample frame of the Federal Statistical Office includes all residential addresses of the Swiss population. To provide a representative picture of the adult population of Switzerland in the survey, a random sample of addresses of adults living in Switzerland was selected according to age categories and language regions within the country. The sample size was calculated on the basis of the required elements for estimating the minimum sample size (e.g., significance level, statistical power level, statistical model used in the DCE analysis and the DCE design), guided by the de Bekker‐Grob et al.'s practical guide [31, 32]. The response rate was 43.8%. The questionnaire was administered online via the survey tool Qualtrics in German, French and Italian to include Switzerland's main language regions. Respondents were excluded if they did not finish the questionnaire (*n* = 157), finished in under 10 min (i.e., half the median time to complete the survey) (*n* = 358) or missed questions on health literacy (*n* = 92). In total, 4745 respondents met these inclusion criteria. The final sample is representative of the general Swiss population for age, sex, health status, nationality and educational background (Supporting Information S1: Appendix B). To check the consistency of the answers, the second choice set was shown twice [33, 34].

### 2.5 Data Analysis

The data analysis was conducted with STATA/MP 17.0. On the basis of the descriptive statistic (percentage distribution of different groups), the baseline characteristics of the sample and the subsamples of the two different DCEs were analysed. The DCEs were analysed using panel latent class logit models (LCA) to determine preference heterogeneity and the relative importance score of the attributes. Using LCA allows the presence and number of unobserved subgroups in the population to be identified on the basis of their preferences. Each respondent has a certain probability of belonging to a class [22, 35]. The alternative specific constant (ASC) controls for any potential bias towards the left or right side of the choice set [36]. The selection of the number of classes in the analysis was guided by the Akaike information criterion (AIC), the Bayesian information criterion (BIC) and the interpretability of the classes [22]. The utility function of the LCA along with the AIC and BIC for different latent classes can be found in Supporting Information S1: Appendices D and E. A class assignment model was estimated to test which respondent characteristics can explain the class assignment of respondents. A better understanding of the latent class membership can be gained using the respondents' characteristics. Based on previous research results, the following characteristics were used to predict class membership: health literacy (measured with the HLS‐EU‐Q6 tool), income (monthly household income in Swiss francs), nationality, age (in years), gender (measured as female and male), satisfaction with current healthcare (self‐rated from 1 to 5), health status (self‐rated from 1 to 5) and education (measured as highest completed education). Supporting Information S1: Appendix C shows the translated questions and response categories for income, gender, age, satisfaction with current healthcare, health status and education. All respondents' characteristics were dummy‐coded in the analysis. Health literacy was categorized as high and low, with high applying to respondents who scored above average on the health literacy questions. People were classified as having high incomes with a monthly net household income above 7000 Swiss Francs, based on the median household income in Switzerland [37]. The variable old was equal to one for people over the age of 65 years. Individuals who rated themselves in the best response category for health status and satisfaction with current healthcare were classified as having a good health status and being satisfied with the current healthcare system. People with a tertiary educational qualification were classified as highly educated (question in Supporting Information S1: Appendix B).

A statistically significant coefficient (*p* < 0.05) indicates that the attribute influenced the participants' decision‐making process. The sign and magnitude of the coefficients show each attribute level's importance compared to its reference level. A positive coefficient indicates that a hypothetical alternative with this attribute level increased utility and a negative coefficient indicates that the attribute level was less desirable than the attribute's reference level. In addition, the relative importance score of each attribute relative to other attributes was estimated overall and for each class by dividing the difference in the utility of the highest and lowest attribute levels of that attribute by the sum of the differences of all attributes. The overall relative importance score was weighted by class sizes. The larger the percentage, the greater the importance relative to the other attributes [38]. Additionally, a sensitivity analysis was conducted, where the results of the respondents who passed the consistency test were estimated and compared with the overall results.

## 3 Results

### 3.1 Respondents

A quarter of the respondents (26%) failed the consistency test included in the DCE. The inconsistency does not necessarily reflect irrational behaviour and may be influenced by learning or fatigue effects [39]. There is a significant correlation between inconsistency, low education and lower health literacy. The results of the consistency test and the overall estimations differ to a certain extent, with no major change to the conclusion. For this research, which has policy implications, it is important to have a representative sample of the population. Therefore, the whole sample was used for further analysis, and the inconsistent answers were not excluded. The final respondents' characteristics are representative of the Swiss population (Supporting Information S1: Appendix B) and are displayed in Table 2. Of the respondents, 22.5% were above 65 years old, 49.6% were male and 29.5% had a high self‐assessed health status. No significant differences were found in the baseline characteristics between the groups answering the different DCEs based on the chi‐squared test (Table 2).

**Table 2:** Baseline characteristics of the final sample for the DCE.

	Total		Acute health problem		Routine examination		*χ* ^2^ test
	*N*	%	*n*	%	*n*	%	*p* value
	4745	100	2441	51.4	2304	48.6	
Age (years)							
Old (≥ 65)	1065	22.5	563	23.1	502	21.8	0.302
Young (< 65)	3676	77.5	1875	76.9	1801	78.2	
Sex							
Male	2348	49.6	1184	48.6	1164	50.6	0.181
Female	2385	50.4	1250	51.4	1135	49.4	
Health status							
High	1397	29.5	713	29.3	684	29.8	0.703
Low	3333	70.5	1720	70.7	1613	70.2	
Nationality							
Swiss	3862	81.4	2025	83.0	1837	79.7	0.356
Not Swiss	883	18.6	415	17.0	468	20.3	
Education							
High (tertiary)	2038	43.0	1013	41.6	1025	44.6	0.100
Primary and secondary	2697	57.0	1423	58.4	1274	55.4	
Income							
High (≥ 7001)	1833	44.3	939	43.9	894	44.8	0.586
Low (< 7001)	2302	55.7	1200	56.1	1102	55.2	
Health literacy							
High (above average)	2491	52.5	1285	52.6	1206	52.3	0.860
Average and below average	2254	47.5	1156	47.4	1098	47.7	
Satisfaction with the current healthcare system							
Very satisfied	853	18.0	419	17.2	434	18.9	0.137
Not very satisfied	3877	82	2016	82.2	1861	81.1	

### 3.2 Latent Class Analysis

The findings of the latent class analysis indicate significant preference heterogeneity in the experiments. The latent class logit models lead to significant improvements in fit compared to the standard logit model. The optimal number of latent classes for the DCE with an acute health problem was two, with class probabilities of 35.6% for Class 1 and 64.4% for Class 2 (Table 3). As shown by the class size, people were likelier to be in Class 2. Specifically, older respondents and respondents with higher incomes as well as those with higher levels of satisfaction with current healthcare, poorer health status and lower education levels were more likely to be in this class. The optimal number of latent classes for the DCE with a routine examination was three, with class probabilities of 34.2% for Class 1, 35.0% for Class 2 and 30.8% for Class 3 (Table 4). Compared to Class 3, respondents in Class 1 were more likely to be immigrants and more likely to be male, older and lower educated. Respondents in Class 2 were older and less educated than respondents in both the other classes. On the basis of their preference patterns, the identified classes are named the money savers (Class 1), the GP appreciators (Class 2) and the ambivalent choosers (Class 3).

**Table 3:** Coefficient table DCE acute health problem.

	Class 1 Money savers Regression coef.^a^	Class 2 GP appreciators Regression coef.
Class probability	35.6%	64.4%
ASC^b^	−0.183*	0.052
First contact (ref. level^c^: general practitioner)	ris^d^ (rank): 10% (4)	25% (1)
Pharmacist	−0.317	−0.852***
Registered nurse	0.228	−0.712***
Healthcare assistant	−0.103	−0.663***
Online application	−0.48*	−0.813***
Type of first contact (ref. level: in persona)	ris (rank): 4% (7)	4% (6)
Via telephone	−0.051	0.010
Via video telephone	0.23	−0.125
Treating person (ref. level: general practitioner)	ris (rank): 6% (6)	21% (2)
Specialized medical doctor	−0.082	−0.070
Registered nurse	−0.26	−0.765***
General practitioner via video telephone + registered nurse	0.162	−0.662***
Opening hours (ref. level: office hours)	ris (rank): 7% (5)	4% (7)
Extended office hours	0.482**	0.039
Around the clock	0.277	0.123*
Continuity (ref. level: knows health h^e^ + access m.r.^f^)	ris (rank): 16% (3)	20% (3)
Does not know health history + access to medical record (m.r.)	−0.374*	−0.233***
Does not know health history, no access to medical record (m.r.)	−1.089***	−0.667***
Decision‐making (ref. level: health professional)	ris (rank): 18% (2)	13% (5)
Patient	1.076***	0.047
Shared decision‐making	1.259***	0.430***
Development monthly health insurance premium (ref. level: stays the same)	ris (rank): 39% (1)	13% (4)
Premium minus 100	0.693***	−0.186*
Premium minus 50	0.413*	−0.330***
Premium plus 50	−0.676***	−0.269***
Premium plus 100	−2.016***	−0.442***
Class assignment model		
Constant	−0.779*	Reference
High health literacy	0.249	Reference
High income	−0.507**	Reference
Swiss	0.075	Reference
Male	−0.197	Reference
Old	−0.560**	Reference
Satisfied with current healthcare	−0.663**	Reference
Good health status	0.458**	Reference
Tertiary education	0.737***	Reference
Model fit		
Observations	23,922	
Log‐likelihood	−6854.815	

*Note:* Based on LCM.

* *p* < 0.05

** *p* < 0.01

*** *p* < 0.001.

^a^ Coefficient.

^b^ Alternative specific constant.

^c^ Reference level.

^d^ Relative importance score.

^e^ Knows health history.

^f^ Access to medical record.

**Table 4:** Coefficient table DCE routine examination.

	Class 1 Money savers Regression coef.^a^	Class 2 GP appreciators Regression coef.	Class 3 Ambivalent choosers Regression coef.
Class probability	30.8%	35.0%	34.2%
ASC^b^	−0.332**	0.271**	−0.045
Treating person (ref. level^c^: General practitioner)	ris^d^ (rank): 10% (3)	20% (2)	11% (5)
Specialized medical doctor	−0.048	−0.665***	0.186
Registered nurse	−0.497	−1.246***	−0.178
General practitioner via videophone + registered nurse	−0.990***	−0.740**	−0.188
Opening hours (ref. level: Office hours)	ris (rank): 5% (7)	9% (6)	13% (4)
Extended office hours	0.535***	−0.245	0.389**
Around the clock	0.137	−0.560***	0.433**
Continuity (ref. level: Knows health h.^e^ + access)	ris (rank): 9% (4)	23% (1)	27% (1)
Does not know health hist. + access to m.r.^f^	−0.022	−0.471**	−0.780***
Does not know health hist., no access to m.r.	−0.880***	−1.469***	−0.895***
Decision‐making (ref. level: Health professional)	ris (rank): 24% (2)	15% (4)	10% (6)
Patient	2.461***	0.212	−0.349
Shared‐decision‐making	2.389***	0.926**	−0.347
Coordination (ref. level: General practitioner)	ris (rank): 9% (5)	18% (3)	4% (7)
Pharmacist	−0.870***	−1.132***	0.063
Registered nurse	−0.447**	−0.428**	−0.066
Healthcare assistant	−0.691**	−0.566***	−0.028
Assignment coordination (ref. level: Patient choice)	ris (rank): 8% (6)	5% (7)	15% (3)
Assignment by health insurance	−0.768***	−0.325*	−0.492***
Development monthly health insurance premium (ref. level: stays the same)	ris (rank): 36% (1)	11% (5)	20% (2)
Premium minus 100	0.992***	−0.047	−0.273
Premium minus 50	1.014***	0.492*	−0.678***
Premium plus 50	−0.369*	0.028	−0.346
Premium plus 100	−2.635***	−0.204	−0.473*
Class assignment model			
Constant	Reference	0.214	0.472
High health literacy	Reference	0.080	0.016
High income	Reference	0.073	0.187
Swiss	Reference	−0.419	−0.684**
Male	Reference	0.223	0.440*
Old	Reference	1.066***	0.558*
Satisfied with current healthcare	Reference	0.327	−0.136
Good health status	Reference	−0.004	0.123
Tertiary education	Reference	−0.509**	−0.448*
Model fit			
Observations	22,580		
Log‐likelihood	−6227.719		

*Note:* Based on LCM.

* *p* < 0.05

** *p* < 0.01

*** *p* < 0.001.

^a^ Coefficient.

^b^ Alternative specific constant.

^c^ Reference level.

^d^ Relative importance score.

^e^ Knows health history.

^f^ Access to medical record.

### 3.3 Coefficients by Latent Classes

Tables 3 and 4 show the coefficients of respondents' preferences for the different levels of the DCE with an acute health problem and routine examination.

For the DCE with an acute health problem (Table 3), all attributes for both classes, except the type of first contact and the treating person for the money savers (Class 1), had statistically significant estimates (*p* < 0.05). For the GP appreciators (Class 2), there was no significant ASC, indicating no significant left or right bias for this class when choosing an alternative in the DCE. The money savers (Class 1) were more likely to select the alternative shown on the right side of the choice set. For first contact, the money savers (Class 1) were indifferent as to whether this task was performed by a GP, pharmacist, registered nurse or healthcare assistant. In contrast, the GP appreciators (Class 2) had a significantly lower preference for all alternatives compared to the GP. Regarding the treating person, again for the money savers (Class 1), it did not matter whether this is a medical doctor (in person or via videophone) or a nurse. For the GP appreciators (Class 2), in‐person treatment by a medical doctor was significantly preferred. Regarding continuity, the money savers (Class 1) and the GP appreciators (Class 2) preferred the treating health professional knowing their health history and having access to their medical records. If the health professional does not know their health history, they should still have access to their medical record. The money savers (Class 1) preferred to be involved in decision‐making regarding the treatment, whether as the primary decision‐maker or through shared decision‐making with the health professional. In contrast, the GP appreciators (Class 2) preferred to be involved in the decision‐making only if the involvement was based on shared decision‐making with the health professional. Regarding the monthly health insurance premium development, the money savers (Class 1) preferred a decrease, whereas the GP appreciators (Class 2) significantly preferred the status quo to any change.

For the DCE with a routine examination (Table 4), all attributes in the classes, except the treating person, decision‐making and coordination person for the ambivalent choosers (Class 3), had statistically significant estimates (*p* < 0.05). For the ambivalent choosers (Class 3), there was no significant ASC, indicating no significant left or right bias for this group when choosing an alternative in the DCE. In contrast, the GP appreciators (Class 2) were more likely to choose the alternative on the left side, and the money savers (Class 1) were more likely to choose the option on the right side. For the treating person, the ambivalent choosers (Class 3) were indifferent as to whether it should be a medical doctor (in person or via videophone) or a nurse (as in the DCE for an acute health problem). For the GP appreciators (Class 2), all alternatives to the GP in person were less preferred. The money savers (Class 1) were indifferent to the educational background of the treating person if no video consultation was involved. For the opening hours, as in the DCE for an acute health problem, the money savers (Class 1) preferred extended office hours and the GP appreciators (Class 2) preferred opening hours around the clock. The ambivalent choosers (Class 3) liked both types of longer opening hours compared to office hours. Regarding continuity, as for the DCE with the acute health problem, the ambivalent choosers (Class 3) and the GP appreciators (Class 2) had a significant preference for the health professional knowing their health history and having access to their medical records. In contrast, the money savers (Class 1) were indifferent about the health professional knowing the health history when access to the medical record was given. Regarding involvement in decision‐making, again, the GP appreciators (Class 2) preferred to be involved in shared decision‐making, and the money savers (Class 1) preferred to be involved even as the primary decision‐makers. Additionally, the ambivalent choosers (Class 3) were indifferent about being involved in decision‐making. Regarding the coordinating health professional, respondents of the ambivalent choosers (Class 3) were unconcerned about the educational background of the coordinating health professional. The GP appreciators (Class 2) and the money savers (Class 1) had significantly lower preference for all options compared to the GP as the coordinating health professional, with the coefficient indicating that a nurse would be the preferred alternative to the GP. For the development of the monthly health insurance premium, only the money savers (Class 1) had a significantly lower preference for the premium being increased and a higher preference for them being decreased. The ambivalent choosers (Class 3) had a lower preference for any change, and the GP appreciators (Class 2) had a higher preference for a decrease of 50 Swiss francs.

### 3.4 Overall Relative Importance Score

**Figure 1:**
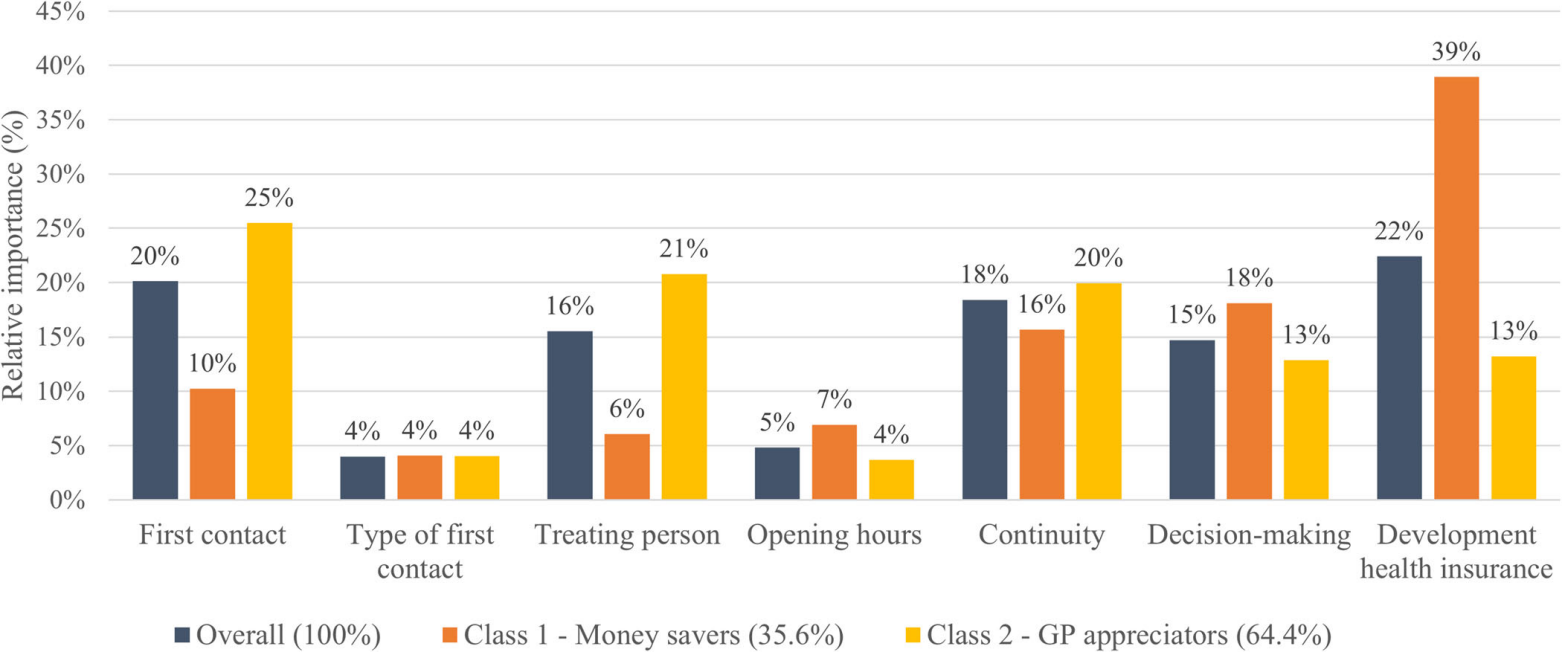
Relative importance score acute health problem.

**Figure 2:**
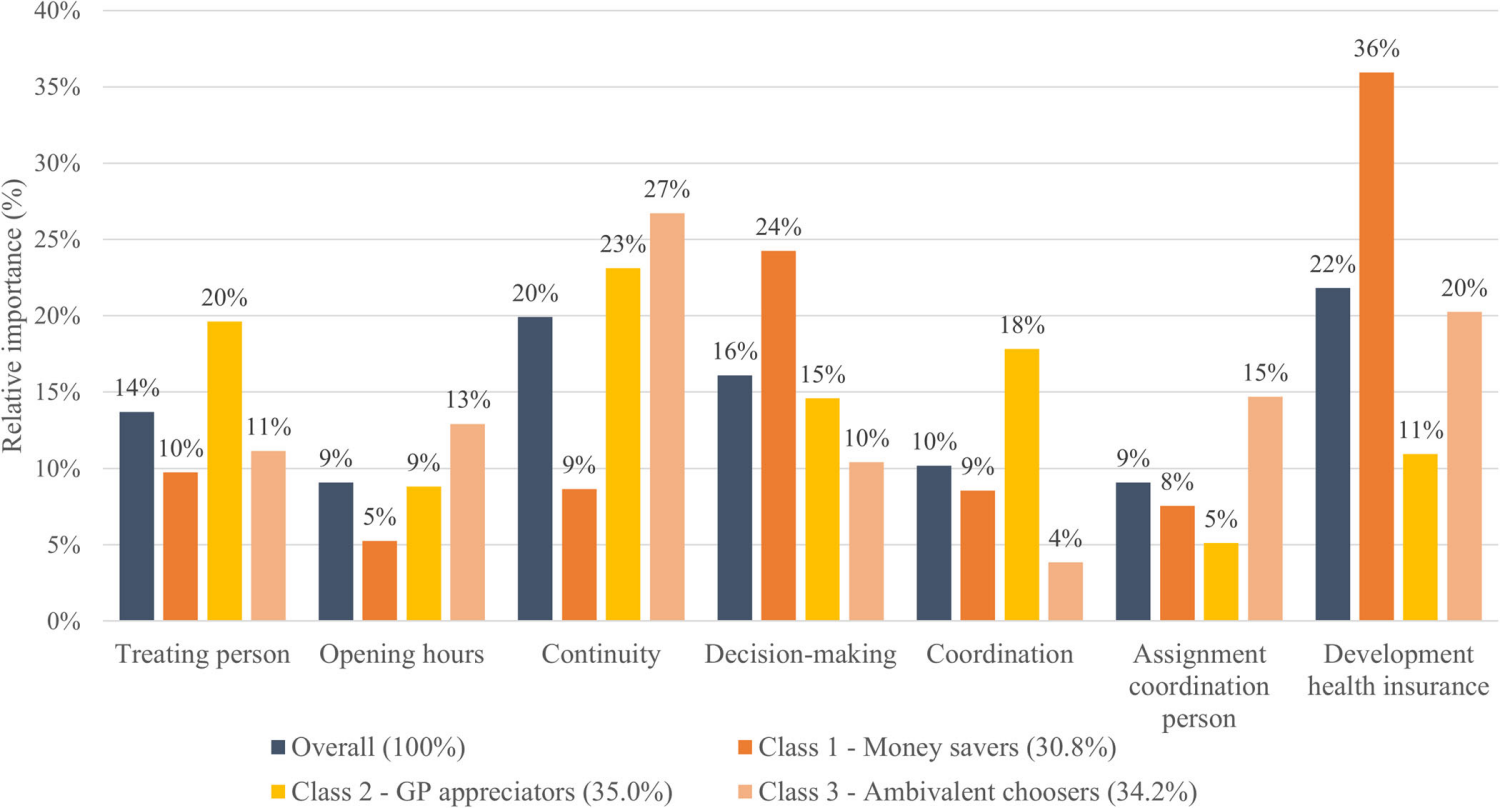
Relative importance score routine examination.

Figures 1 and 2 show the relative importance score of the attributes when choosing an alternative in the DCE. The overall results for the DCE including an acute health problem (Figure 1) indicate that the development of health insurance had the highest relative importance score (22%), followed by first contact (20%) and continuity of the health professional (18%). In contrast, opening hours (5%) and type of first contact (4%) had the lowest relative importance score overall. For the DCE including a routine examination (Figure 2), the development of health insurance premium was again the most important attribute for the respondents in relation to all other attributes (22%), closely followed by the continuity of the health professional (20%) and the respondents' engagement in decision‐making (16%). Opening hours (9%) and the assignment of the coordinating person (9%) were the attributes with the lowest relative importance score.

### 3.5 Relative Importance Score by Latent Classes

Latent classes differ in their preference of attributes, as seen in differences in the ranking in the relative importance score. For the DCE with the acute health problem, the GP appreciators (Class 2) assigned the highest relative importance score to the first contact (25%), followed by the treating person (21%) and the continuity (20%). For this class, the development of health insurance had a comparably low relative importance score (13%). In contrast, for the money savers (Class 1), the development of health insurance had the highest relative importance score (39%), followed by the involvement in decision‐making (18%). The same pattern was estimated for the DCE with a routine examination: the GP appreciators (Class 2) assigned high relative importance to the continuity (23%) as well as the education of the health professionals (treating person 20%, coordinating health professional 18%) and relative minor importance to the development of the health insurance (11%). At the same time, the money savers (Class 1) assigned the highest relative importance to the development of health insurance (36%) and the involvement in decision‐making (24%). The ambivalent choosers (Class 3) of the DCE with a routine examination also assigned the highest relative importance to the continuity (27%). In comparison to the other two classes, this class assigned a high relative importance score to the assignment of the coordinating health professional (15%) and the opening hours (13%) and a relatively low relative importance to the decision‐making (10%) and the coordinating health professional (4%).

## 4 Discussion

The overall findings indicate that the population places high value on an outpatient primary healthcare system with continuity of the health professional in charge. This finding on a preference of the population for a long‐term relationship with their health professionals is in line with previous research [40, 41, 42]. The results indicate that this attribute is essential for both an acute health problem and a routine examination but is especially important when routine examinations are performed. These findings align with previous research, which found that respondents were willing to wait longer to see a familiar doctor for a routine check‐up than for a visit with a minor health problem [43]. In contrast, multiple studies indicate that in Western countries such as the United States and England, continuity of care is declining [44, 45]. The reasons for this are diverse, such as a lack of available GPs, changing work structures or a trend towards larger practices and healthcare centres with a larger team serving a more extensive patient list [44, 46, 47]. In line with previous research [44, 47], this study suggests that it is important to invest in and develop further strategies to ensure continuity of care in future healthcare to meet patients' preferences [44, 47]. If personal continuity of care is not possible, an electronic patient dossier is one way to still ensure informational continuity. The preference for health professionals to have access to the medical records, even when they do not know the patient, emphasizes the population's acceptance of electronic patient dossiers. Another development that contributes to meeting the need for continuity of care despite the lack of GP is the use of telehealth. Here, the results indicate that, overall, relatively little importance is placed on whether the first contact is done in person or via (video)phone. This suggests that telehealth services could be a widely accepted development for non‐emergency health problems.

The findings further indicate significant preference heterogeneity for current developments in outpatient primary healthcare. The latent class logit models lead to significant improvements in fit compared to the standard logit model. The LCA revealed two different choice patterns for outpatient primary healthcare in the DCE with an acute health problem and three for the DCE with a routine examination.

The results find that for one part of the population (the GP appreciators), the educational background of the health professionals involved in their outpatient health visit is essential. For them, it is important that the first contact, treatment and coordination are provided by a GP. They also attach a high relative importance to personal and informational continuity, as it is the case with today's role of the GP in Switzerland. A class with these preference structures can be identified for both the acute situation (64.4% of respondents) and the routine examination (35.0% of respondents). In contrast, this preference class does not attach high relevance to the development of insurance premium, indicating that they are willing to pay more to get their preferred options in a healthcare visit. The group of respondents with this preference structure is more likely to be old and satisfied with current healthcare and less likely to have a tertiary education and be in good health than the respondents of the other preference classes. The finding is consistent with previous research, which has found that older people more often cite qualifications as a reason for their preference for primary care provider type than younger people [48]. The introduction of advanced roles for nurses often focuses on managing patients with chronic conditions, concerning mainly older patients [49]; accordingly, this preference can impede the success of advanced nurse practitioner care models. As, in addition to age, education level is also related to openness to receive care from non‐medical health professionals, education on the competencies of non‐medical health professionals could help to increase the acceptance of task shifting in the population.

The results further indicate the existence of a preference class (the money savers) that attaches low importance to the educational background of the health professional. The results indicate that for this class, it is not relevant to have a medical doctor taking over first contact and treatment, but that they are open to a registered nurse taking over this task. However, for this group, the development of the health insurance premium has high relative importance. They attach great importance to the health insurance premium not becoming more expensive. Furthermore, they necessarily want to be involved in decision‐making. Lastly, this preference class attaches importance to continuity. But other than the first preference class discussed, not on personal continuity, but mostly on informational continuity. Respondents with this preference structure are more likely to be young, have a tertiary education, be Swiss and female and less likely to have a high income than the respondents with the other preference structures. Again, this preference pattern was observed for both the acute situation (35.6% of the respondents) as well as the routine examination (30.8% of the respondents). This result indicates an openness of this part of the population to task shifting from a doctor to a nurse, particularly if health insurance premiums can be saved as a result. In countries with an insurance system that allows the choice of managed care plans, such as the United States [50] or Switzerland [51], this could be implemented by completing the existing plans with a scheme in which a nurse practitioner takes over certain tasks traditionally performed by the GP. By choosing this model, patients pay lower premiums. However, the result also indicates that from a health policy's point of view, it is of little use to offer those cost‐oriented insurance models because older people who cause high costs are less likely to respond to them. This form of financial incentives is therefore unlikely to reduce healthcare costs. On the basis of these results, a more effective approach to increase acceptance of other health professionals taking over tasks from doctors could be to invest in education on the topic.

For the routine examination, a third group (the ambivalent choosers) with a distinct preference structure was observed (34.2% of the respondents). This group attaches high relative importance to personal continuity, high relative importance on healthcare premium not changing (in both directions), and significantly more importance to the self‐choice for the coordinating health professional and for longer opening hours than the other preference classes. At the same time, they do not attach a high level of relevance to the educational background of the health professionals and on participation in medical decision‐making. Thus, instead of the GP, they would likely accept other health professionals such as a registered nurse as their main healthcare provider. Respondents of this preferences class are less likely Swiss and satisfied with current healthcare and more likely male than the respondents of the other preference classes.

Although the study's results are individually consistent with previous research, most existing studies have focused on one trend of primary outpatient healthcare. The main strength of this study is that it covers several aspects currently subject to discussion within one experiment, allowing for a comparison of their relevance and identifying patterns regarding preferences for future healthcare. However, this study has several limitations. First, the analysed data refer exclusively to the Swiss context. Preferences might differ in populations of other states, as how current outpatient primary healthcare is organized differs between countries and healthcare systems. Therefore, future research should seek to confirm these reported preferences in other countries with healthcare systems comparable to Switzerland. Second, further research should examine health professionals' preferences for developments in outpatient primary healthcare, as future changes to healthcare provision need to be supported by both patients and professionals to ensure that working in healthcare remains attractive for health professionals. A third limitation of the presented study is the different colours used in the choice sets of the DCEs. This could have had an unintended influence on the respondents' choices [52]. Additionally, the preference indicated in the study may differ from the revealed preference. However, previous studies have shown that DCEs can produce results with reasonable external validity [53].

## 5 Conclusion

In summary, the findings indicate that there are heterogeneous preferences of the general population for current developments in outpatient primary healthcare such as task shifting, e‐health, continuity of the health professional, shared decision‐making and healthcare cost. For older and less educated people, having a medical doctor for all healthcare tasks and personal continuity with this healthcare professional plays a crucial role in the choice of primary healthcare models. Low health insurance premiums and participation in medical decisions are most important for younger and more educated people. For this part of the population, informational continuity of health information is more important than personal continuity. According to the study, this part of the population is more willing to accept current trends, such as task shifting, if they can lower their health insurance premiums in return. The population with this preference structure might also be more open to further pilot projects in healthcare. For the other part of the population, moving away from the status quo in terms of the educational background of the health professional and personal continuity is not compatible with their acceptance of the health system. This must be considered in the further development of the health system, preferably through a selection of different options. However, education and awareness raising for these trends could be a source to increase acceptance.

## Author Contributions

**Zora Föhn:** conceptualization, investigation, writing–original draft, methodology, visualization, writing–review and editing, software, formal analysis, and data curation.

## Ethics Statement

This study provides Clarification of responsibility of the Ethics Committee Northwest and Central Switzerland (Req‐2020‐00988).

## Conflicts of Interest

The author declares no conflicts of interest.

## Supporting information

Supporting information.

## Data Availability

The data that support the findings of this study are available from the corresponding author upon reasonable request.

## References

[1] “Health at a Glance: OECD Indicators,” OECD, 2019, 10.1787/4dd50c09-en.

[2] A. Chłoń‑Domińczak , I. E. Kotowska , J. Kurkiewicz , M. Stonawski , and A. Abramowska‑Kmon , Population Ageing in Europe. Facts, Implications and Policies (Brussels: EUROPEAN COMMISSION, Directorate‐General for Research and Innovation, 2014), 10.13140/2.1.5039.6806.

[3] J. Stokes , M. Panagioti , R. Alam , K. Checkland , S. Cheraghi‐Sohi , and P. Bower , “Effectiveness of Case Management for ‘At Risk’ Patients in Primary Care: A Systematic Review and Meta‐Analysis,” PLoS One 10 7 (2015): e0132340, 10.1371/journal.pone.0132340. 26186598 PMC4505905

[4] J. Abraham , B. Whiteman , J. Coad , and R. Kneafsey , “Development and Implementation of Non‐Medical Practitioners in Acute Care,” British Journal of Nursing 25, no. 20 (2016): 1129–1134, 10.12968/bjon.2016.25.20.1129. 27834513

[5] D. S. Kringos , W. G. W. Boerma , A. Hutchinson , R. B. Saltman , and European Observatory on Health Systems and Policies , Building Primary Care in a Changing Europe (Copenhagen: WHO Regional Office for Europe, 2015), https://www.ncbi.nlm.nih.gov/books/NBK459010/. 29035488

[6] A. Eccles , M. Hopper , A. Turk , and H. Atherton , “Patient Use of an Online Triage Platform: A Mixed‐Methods Retrospective Exploration in UK Primary Care,” British Journal of General Practice 69, no. 682 (2019): e336–e344, 10.3399/bjgp19X702197. PMC6478479 30910874

[7] M. A. Smith , “The Role of Shared Decision Making in Patient‐Centered Care and Orthopaedics,” Orthopaedic Nursing 35, no. 3 (2016): 144–149, 10.1097/NOR.0000000000000243. 27187217

[8] K. S. Kleij , U. Tangermann , V. E. Amelung , and C. Krauth , “Patients' Preferences for Primary Health Care—A Systematic Literature Review of Discrete Choice Experiments,” BMC Health Services Research 17, no. 1 (2017): 476, 10.1186/s12913-017-2433-7. 28697796 PMC5505038

[9] V. Soekhai , E. W. de Bekker‐Grob , A. R. Ellis , and C. M. Vass , “Discrete Choice Experiments in Health Economics: Past, Present and Future,” PharmacoEconomics 37, no. 2 (2019): 201–226, 10.1007/s40273-018-0734-2. 30392040 PMC6386055

[10] S. Cheraghi‐Sohi , A. R. Hole , N. Mead , et al., “What Patients Want From Primary Care Consultations: A Discrete Choice Experiment to Identify Patients' Priorities,” Annals of Family Medicine 6, no. 2 (2008): 107–115, 10.1370/afm.816. 18332402 PMC2267425

[11] M. F. Longo , D. R. Cohen , K. Hood , et al., “Involving Patients in Primary Care Consultations: Assessing Preferences Using Discrete Choice Experiments,” British Journal of General Practice: The Journal of the Royal College of General Practitioners 56, no. 522 (2006): 35–42. 16438813 PMC1821413

[12] K. Gerard , V. Lattimer , H. Surridge , et al., “The Introduction of Integrated Out‐of‐Hours Arrangements in England: A Discrete Choice Experiment of Public Preferences for Alternative Models of Care,” Health Expectations 9, no. 1 (2006): 60–69, 10.1111/j.1369-7625.2006.00365.x. 16436162 PMC5060322

[13] J. Caldow , C. Bond , M. Ryan , et al., “Treatment of Minor Illness in Primary Care: A National Survey of Patient Satisfaction, Attitudes and Preferences Regarding a Wider Nursing Role,” Health Expectations 10, no. 1 (2007): 30–45, 10.1111/j.1369-7625.2006.00422.x. 17324193 PMC5060381

[14] K. Gerard , M. Tinelli , S. Latter , A. Smith , and A. Blenkinsopp , “Patients' Valuation of the Prescribing Nurse in Primary Care: A Discrete Choice Experiment,” Health Expectations 18, no. 6 (2015): 2223–2235, 10.1111/hex.12193. 24720861 PMC5810682

[15] M. Droz , N. Senn , and C. Cohidon , “Communication, Continuity and Coordination of Care Are the Most Important Patients' Values for Family Medicine in a Fee‐for‐Services Health System,” BMC Family Practice 20, no. 1 (2019): 19, 10.1186/s12875-018-0895-2. 30683051 PMC6346577

[16] H. P. Jung , C. Baerveldt , F. Olesen , R. Grol , and M. Wensing , “Patient Characteristics as Predictors of Primary Health Care Preferences: A Systematic Literature Analysis,” Health Expectations 6, no. 2 (2003): 160–181, 10.1046/j.1369-6513.2003.00221.x. 12752744 PMC5060177

[17] J. M. Polinski , T. Barker , N. Gagliano , A. Sussman , T. A. Brennan , and W. H. Shrank , “Patients' Satisfaction With and Preference for Telehealth Visits,” Journal of General Internal Medicine 31, no. 3 (2016): 269–275, 10.1007/s11606-015-3489-x. 26269131 PMC4762824

[18] M. K. Kaltoft , R. Turner , M. Cunich , G. Salkeld , J. B. Nielsen , and J. Dowie , “Addressing Preference Heterogeneity in Public Health Policy by Combining Cluster Analysis and Multi‐Criteria Decision Analysis: Proof of Method,” Health Economics Review 5, no. 1 (2015): 10, 10.1186/s13561-015-0048-4. 25992305 PMC4429422

[19] M. Ryan , “Discrete Choice Experiments in Health Care,” BMJ 328, no. 7436 (2004): 360–361, 10.1136/bmj.328.7436.360. 14962852 PMC341374

[20] W. Adamowicz , P. Boxall , M. Williams , and J. Louviere , “Stated Preference Approaches for Measuring Passive Use Values: Choice Experiments and Contingent Valuation,” American Journal of Agricultural Economics 80, no. 1 (1998): 64–75, 10.2307/3180269.

[21] D. McFadden , “Econometric Models of Probabilistic Choice,” Structural Analysis of Discrete Data With Econometric Applications (MIT Press, 1981), 198–272.

[22] A. B. Hauber , J. M. González , C. G. M. Groothuis‐Oudshoorn , et al., “Statistical Methods for the Analysis of Discrete Choice Experiments: A Report of the ISPOR Conjoint Analysis Good Research Practices Task Force,” Value in Health 19, no. 4 (2016): 300–315, 10.1016/j.jval.2016.04.004. 27325321

[23] J. J. Louviere , D. A. Hensher , J. D. Swait , and W. Adamowicz , Stated Choice Methods: Analysis and Applications, 1st ed. (Cambridge University Press, 2000), 10.1017/CBO9780511753831.

[24] M. Ryan , V. Watson , and V. Entwistle , “Rationalising the ‘Irrational’: A Think Aloud Study of Discrete Choice Experiment Responses,” Health Economics 18, no. 3 (2009): 321–336, 10.1002/hec.1369. 18651601

[25] A. Balthasar , A. Hanimann , and C. Strotz , “Ambulante Medizinische Grund‐Versorgung 2040: Einstellungen Und Präferenzen von Patienten/‐Innen Und Health Professinals,” Interface Politikstudien Forschung Beratung, 2017.

[26] A. De Brún , D. Flynn , L. Ternent , et al., “A Novel Design Process for Selection of Attributes for Inclusion in Discrete Choice Experiments: Case Study Exploring Variation in Clinical Decision‐Making About Thrombolysis in the Treatment of Acute Ischaemic Stroke,” BMC Health Services Research 18, no. 1 (2018): 483, 10.1186/s12913-018-3305-5. 29929523 PMC6013945

[27] J. R. DeShazo and G. Fermo , “Designing Choice Sets for Stated Preference Methods: The Effects of Complexity on Choice Consistency,” Journal of Environmental Economics and Management 44, no. 1 (2002): 123–143, 10.1006/jeem.2001.1199.

[28] S. N. Settumba , M. Shanahan , T. Butler , et al., “Developing Attributes and Attribute‐Levels for a Discrete‐Choice Experiment: An Example for Interventions of Impulsive Violent Offenders,” Applied Health Economics and Health Policy 17, no. 5 (2019): 683–705, 10.1007/s40258-019-00484-5. 31161367

[29] C. B. Kamphuis , E. W. de Bekker‐Grob , and F. J. van Lenthe , “Factors Affecting Food Choices of Older Adults From High and Low Socioeconomic Groups: A Discrete Choice Experiment,” American Journal of Clinical Nutrition 101, no. 4 (2015): 768–774, 10.3945/ajcn.114.096776. 25833974

[30] “Ngene 1.2: User Manual & Reference Guide,” ChoiceMetrics, Published online 2018, http://www.choice-metrics.com/NgeneManual120.pdf.

[31] E. W. de Bekker‐Grob , B. Donkers , M. F. Jonker , and E. A. Stolk , “Sample Size Requirements for Discrete‐Choice Experiments in Healthcare: A Practical Guide,” Patient‐Patient‐Centered Outcomes Research 8, no. 5 (2015): 373–384, 10.1007/s40271-015-0118-z. 25726010 PMC4575371

[32] V. Watson , F. Becker , and E. de Bekker‐Grob , “Discrete Choice Experiment Response Rates: A Meta‐Analysis: Discrete Choice Experiment Response Rates: A Meta‐Analysis,” Health Economics 26, no. 6 (2017): 810–817, 10.1002/hec.3354. 27122445

[33] R. Brouwer , I. Logar , and O. Sheremet , “Choice Consistency and Preference Stability in Test‐Retests of Discrete Choice Experiment and Open‐Ended Willingness to Pay Elicitation Formats,” Environmental and Resource Economics 68, no. 3 (2017): 729–751, 10.1007/s10640-016-0045-z.

[34] F. R. Johnson , J. C. Yang , and S. D. Reed , “The Internal Validity of Discrete Choice Experiment Data: A Testing Tool for Quantitative Assessments,” Value in Health 22, no. 2 (2019): 157–160, 10.1016/j.jval.2018.07.876. 30711059

[35] I. C. Wurpts and C. Geiser , “Is Adding More Indicators to a Latent Class Analysis Beneficial or Detrimental? Results of a Monte‐Carlo Study,” Frontiers in Psychology 5 (2014), 10.3389/fpsyg.2014.00920. PMC4140387 25191298

[36] I. Mokas , S. Lizin , T. Brijs , N. Witters , and R. Malina , “Can Immersive Virtual Reality Increase Respondents' Certainty in Discrete Choice Experiments? A Comparison With Traditional Presentation Formats,” Journal of Environmental Economics and Management 109 (2021): 102509, 10.1016/j.jeem.2021.102509.

[37] “Haushaltsbudgeterhebung: Ergebnisse,” BFS, Published online 2019, https://www.bfs.admin.ch/bfsstatic/dam/assets/19264887/master.

[38] I. G. Arslan , S. P. I. Huls , E. W. de Bekker‐Grob , et al., “Patients', Healthcare Providers', and Insurance Company Employees' Preferences for Knee and Hip Osteoarthritis Care: A Discrete Choice Experiment,” Osteoarthritis and Cartilage 28, no. 10 (2020): 1316–1324, 10.1016/j.joca.2020.07.002. 32682071

[39] S. Özdemir , A. F. Mohamed , F. R. Johnson , and A. B. Hauber , “Who Pays Attention in Stated‐Choice Surveys?,” Health Economics 19, no. 1 (2010): 111–118, 10.1002/hec.1452. 19267358

[40] K. M. Ehman , M. Deyo‐Svendsen , Z. Merten , A. M. Kramlinger , and G. M. Garrison , “How Preferences for Continuity and Access Differ Between Multimorbidity and Healthy Patients in a Team Care Setting,” Journal of Primary Care & Community Health 8, no. 4 (2017): 319–323, 10.1177/2150131917704556. PMC5932726 28434390

[41] C. Liu , Y. Wu , and X. Chi , “Relationship Preferences and Experience of Primary Care Patients in Continuity of Care: A Case Study in Beijing, China,” BMC Health Services Research 17, no. 1 (2017): 585, 10.1186/s12913-017-2536-1. 28830507 PMC5568350

[42] R. Baker , M. Boulton , K. Windridge , C. Tarrant , J. Bankart , and G. K. Freeman , “Interpersonal Continuity of Care: A Cross‐Sectional Survey of Primary Care Patients' Preferences and Their Experiences,” British Journal of General Practice: The Journal of the Royal College of General Practitioners 57, no. 537 (2007): 283–289. 17394731 PMC2043338

[43] D. Turner , C. Tarrant , K. Windridge , et al., “Do Patients Value Continuity of Care in General Practice? An Investigation Using Stated Preference Discrete Choice Experiments,” Journal of Health Services Research & Policy 12, no. 3 (2007): 132–137, 10.1258/135581907781543021. 17716414

[44] I. Barker , A. Steventon , and S. R. Deeny , “Association Between Continuity of Care in General Practice and Hospital Admissions for Ambulatory Care Sensitive Conditions: Cross Sectional Study of Routinely Collected, Person Level Data,” BMJ 356 (2017): j84, 10.1136/bmj.j84. 28148478

[45] P. Tammes , R. W. Morris , M. Murphy , and C. Salisbury , “Is Continuity of Primary Care Declining in England? Practice‐Level Longitudinal Study From 2012 to 2017,” British Journal of General Practice 71, no. 707 (2021): e432–e440, 10.3399/BJGP.2020.0935. PMC8103927 33947666

[46] M. Marshall and D. P. Gray , “General Practice Is Making a Leap in the Dark,” BMJ 355 (2016): i5698, 10.1136/bmj.i5698. 27793815

[47] H. Jeffers and M. Baker , “Continuity of Care: Still Important in Modern‐Day General Practice,” British Journal of General Practice 66, no. 649 (2016): 396–397, 10.3399/bjgp16X686185. PMC4979920 27481958

[48] B. Leach , M. Gradison , P. Morgan , C. Everett , M. J. Dill , and J. S. de Oliveira , “Patient Preference in Primary Care Provider Type,” Healthcare 6, no. 1 (2018): 13–16, 10.1016/j.hjdsi.2017.01.001. 28602803

[49] C. Maier , L. Aiken , and R. Busse , “Nurses in Advanced Roles in Primary Care: Policy Levers for Implementation,” 98, 2017, 10.1787/a8756593-en.

[50] T. Rice , P. Rosenau , L. Y. Unruh , and A. J. Barnes , “United States: Health System Review,” Health Systems in Transition 22, no. 4 (2020): 1–441. 33527901

[51] C. De Pietro , P. Camenzind , I. Sturny , et al., “Switzerland: Health System Review,” Health Systems in Transition 17, no. 4 (2015): 1–288. 26766626

[52] M. F. Jonker , B. Donkers , E. W. de Bekker‐Grob , and E. A. Stolk , “Effect of Level Overlap and Color Coding on Attribute Non‐Attendance in Discrete Choice Experiments,” Value in Health 21, no. 7 (2018): 767–771, 10.1016/j.jval.2017.10.002. 30005748

[53] M. Quaife , F. Terris‐Prestholt , G. L. Di Tanna , and P. Vickerman , “How Well Do Discrete Choice Experiments Predict Health Choices? A Systematic Review and Meta‐Analysis of External Validity,” European Journal of Health Economics 19, no. 8 (2018): 1053–1066, 10.1007/s10198-018-0954-6. 29380229

