# Supplementary material for: Task Shifting, eHealth and Shared Decision‐Making—Preference Heterogeneity in the Adult Population for Developments in Outpatient Primary Healthcare
Source: Health Expect. 2025 Feb 5;28(1):e70060. doi: 10.1111/hex.70060 (PMC11795417; doi:10.1111/hex.70060)
Supplement: Supplementary file 1 — Supporting information. [file HEX-28-e70060-s001.docx]

**Appendix**

1. Example of Choice Set in the survey (translated to English)


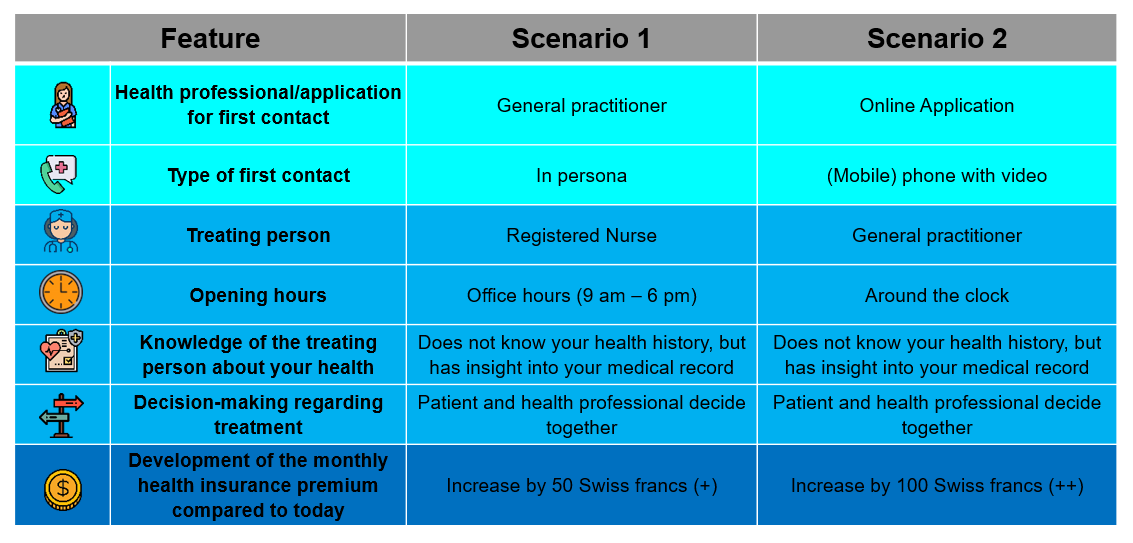


Which of the scenarios would you prefer for the situation described in 2040?

1. Sample description

Comparison of baseline characteristics of sample with Swiss adult population

|  | | | Sample | Swiss population |
| --- | --- | --- | --- | --- |
| **Age** | | |  |  |
|  | Old (≥ 65 years) | | 22.5% | 22.0% (bfs,2019) |
| **Sex** | | |  |  |
|  | | Male | 49.6% | 49.6% (bfs, 2021) |
| **Health status** | | |  |  |
|  | | Good or very good | 82.9% | 84.7% (Swiss Health Survey 2017) |
| **Nationality** | | |  |  |
|  | | Swiss | 81.4% | 75.0% (bfs, 2021) |
| **Education** | | |  |  |
|  | | High (Tertiary) | 43.0% | 45.3% (bfs, 2020) |

1. Questions in the survey for membership variables

| Variable | Question (translated to English) | Response categories |
| --- | --- | --- |
| **Income** | If you add up the income from all sources, what is the total net income (salary paid after deductions for AHV, IV, pension fund, alimony, pensions, scholarships, unemployment benefits, etc.) of your household per month?  Please give an estimate if you do not know the exact figures. | - < 3’000.- - 3’001- 5’000.- - 5’001- 7’000.- - 7’001- 9’000.- - 9’001- 11’000.- - 11’001- 13’000.- - > 13’000.- |
| **Gender** | Please state your gender. | - Male - Female |
| **Age** | Please state your year of birth. |  |
| **Satisfaction with current healthcare** | In general, how satisfied or dissatisfied are you with the Swiss healthcare system? | - Very dissatisfied - Rather dissatisfied - Neither satisfied nor dissatisfied - Rather satisfied - Very satisfied |
| **Health status** | How is your health in general? | - Very poor - Poor - Average - Good - Very good |
| **Education** | What is your highest completed education? | - No completed school - Compulsory school - Vocational apprenticeship or vocational school - Grammar School - Higher technical and vocational training - Higher technical college - University, university of applied sciences - Don't know |

1. Utility function
   1. DCE with an acute health problem

V_rta|c =_ β 0|c + β1_|_c First contact _Pharmacist_ *_rta|c_* + β2_|_c First contact _Registered nurse_ *_rta|c_* + β3_|_c First contact _Healthcare assistant_ *_rta|c_* + β4_|_c First contact _Online application_ *_rta|c_* + β5_|_c Type of first contact _Via videophone_ *_rta|c_* + β6_|_c Type of first contact _Via phone_ *_rta|c c_* + β7_|_c Treating person _Specialised medical practitioner_ *_rta|c_* + β8_|_c Treating person _Registered nurse_ *_rta|c_* + β9_|_c Treating person _GP via videophone_ *_rta|c_* + β10_|_c Opening hours _Extended office hours_ *_rta|c_* + β11_|_c Opening hours _Around-the-clock_ *_rta|c_* + β12_|_c Continuity _No knowledge + Access_ *_rta|c_* + β13_|_c Continuity _No knowledge, no access_ *_rta|c_* + β14_|_c Decision-making _patient_ *_rta|c c_* + β15_|_c Decision-making _shared_ *_rta|c_* + β16_|_c Development health insurance _-100.-_ *_rta|c_* + β17_|_c Development health insurance _-50.-_ *_rta|c_* + β18_|_c Development health insurance _+50.-_ *_rta|c_* + β19_|_c Development health insurance _+100.-_ *_rta|c_*

Where

- V_rta|c_ represent the observable utility that respondent r belonging to class c reported for alternative a in choice task t.
- b0|c represents an alternative-specific constant for a certain class
- b1-19|c represent the attribute-level estimates that indicate the relative importance of each attribute level
  1. Utility function DCE with a routine examination

V_rta|c =_ β 0|c + β1_|_c Treating person _Specialised medical practitioner_ *_rta|c_* + β2_|_c Treating person _Registered nurse_ *_rta|c_* + β3_|_c Treating person _GP via videophone_ *_rta|c_* + β4_|_c Opening hours _Extended office hours_ *_rta|c_* + β5_|_c Opening hours _Around-the-clock_ *_rta|c_* + β6_|_c Continuity _No knowledge + Access_ *_rta|c_* + β7_|_c Continuity _No knowledge, no access_ *_rta|c_* + β8_|_c Decision-making _patient_ *_rta|c c_* + β9_|_c Decision-making _shared_ *_rta|c_* + β10_|_c Coordinating person _Pharmacist_ *_rta|c_* + β11_|_c Coordinating person _Registered nurse_ *_rta|c_* + β12_|_c Coordinating person _Healthcare assistant_ *_rta|c_* + β13_|_c Assignment coordinating person _By health insurance_ *_rta|c_* +β14_|_c Development health insurance _-100.-_ *_rta|c_* + β15_|_c Development health insurance _-50.-_ *_rta|c_* + β16_|_c Development health insurance _+50.-_ *_rta|c_* + β17_|_c Development health insurance _+100.-_ *_rta|c_*

Where

- V_rta|c_ represent the observable utility that respondent r belonging to class c reported for alternative a in choice task t.
- b0|c represents an alternative-specific constant for a certain class
- b1-17|c represent the attribute-level estimates that indicate the relative importance of each attribute level

1. AIC and BIC for different latent classes
   1. DCE with an acute health problem
   2. DCE with a routine examination
